# Light attenuates lipid accumulation while enhancing cell proliferation and starch synthesis in the glucose-fed oleaginous microalga *Chlorella zofingiensis*

**DOI:** 10.1038/srep14936

**Published:** 2015-10-07

**Authors:** Tianpeng Chen, Jin Liu, Bingbing Guo, Xiaonian Ma, Peipei Sun, Bin Liu, Feng Chen

**Affiliations:** 1 Institute for Food & Bioresource Engineering, College of Engineering, Peking University, Beijing, 100871, China

## Abstract

The objective of this study was to investigate the effect of light on lipid and starch accumulation in the oleaginous green algae *Chlorella zofingiensis* supplemented with glucose. *C. zofingiensis*, when fed with 30 g/L glucose, synthesized lipids up to 0.531 g/g dry weight; while in the presence of light, the lipid content dropped down to 0.352 g/g dry weight. Lipid yield on glucose was 0.184 g/g glucose, 14% higher than that cultured with light. The light-mediated lipid reduction was accompanied by the down-regulation of fatty acid biosynthetic genes at the transcriptional level. Furthermore, light promoted cell proliferation, starch accumulation, and the starch yield based on glucose. Taken together, light may attenuate lipid accumulation, possibly through the inhibition of lipid biosynthetic pathway, leading to more carbon flux from glucose to starch. This study reveals the dual effects of light on the sugar-fed *C. zofingiensis* and provides valuable insights into the possible optimization of algal biomass and lipid production by manipulation of culture conditions.

It is commonly agreed that environmental deterioration and fossil fuel depletion are becoming two worldwide issues that threaten human development. Other energy sources that are green and renewable have been sought over the past several decades, and biofuel has substantial potential to be such an alternative. Biodiesel, composed of fatty acid methyl esters (FAMEs), is regarded as a favorable biofuel because of its sustainability, non-toxicity, and adaptability for existing engines^1^. Currently, vegetable oils such as soybean, oil palm, jatropha, and coconut are the main sources for commercial biodiesel production^2^,^3^. However, there are concerns over land occupation for biodiesel production by oil plants because it would threaten the food supply, increase deforestation rates and damage biodiversity^4^. Therefore, alternative feedstocks for biodiesel are sought after.

Oleaginous microalgae have been suggested as potential biodiesel-producing candidates because of many advantages such as fast growth, high oil productivity, and low arable land demand^5^,^6^. In addition, biodiesel from microalgal oil is superior to the standard biodiesel because it is more stable in terms of flash point values while the other properties are similar to the standard biodiesel^7^. Moreover, microalgae cultivation in bioreactors can be better controlled making it suitable for industrial production.

At present, research has focused on the photoautotrophic production of microalgal oil using light as the energy source. But there are significant drawbacks associated with photoautotrophic algal cultures for oil production. First, it is difficult to solve the contradiction between accumulation of biomass and lipid synthesis during the microalgal life cycle^8^. Second, light attenuation is unavoidable for photoautotrophic cultures from lab to pilot scale, and it may significantly reduce productivity^9^. Some microalgal species, such as *Chlorella* spp., can grow faster and accumulate biomass more efficiently when utilizing an organic carbon source than photosynthesis, leading to ultrahigh cell density and biomass productivity^10^. Thus, the above mentioned shortcomings can be eliminated by culturing algae with organic carbon source in the dark, and the high productivity will to some extent offset the cost of organic carbon.

Glucose is the most commonly used organic carbon source for microalgae cultivation. Microalgae cultured with glucose may have far higher growth and respiration rates than that with such other organic carbons as acetate, galactose, and fructose, mainly due to the fact that glucose has more energy per mol and is able to affect metabolic pathways involving carbon assimilation, cell size, and starch and lipid synthesis^11^,^12^. Interestingly, microalgae grow in quite different manners with and without glucose or light^13^,^14^. This might be explained by the fact that glucose is mainly metabolized via the pentose phosphate pathway (PPP), which generates more cellular ATP and NADPH in the dark than the glycolytic pathway in the light, according to previous reports^15^,^16^,^17^. NADPH is required as reducing powder for the biosynthesis of fatty acids and lipids^18^. However, there is no explicit data about the differences in cell proliferation and energy substance accumulation between the cultures with and without light. Data are also lacking about the lipid yield on glucose, which is an important cost factor for algal oil production.

*Chlorella zofingiensis* is a green alga that can grow well photoautotrophically and on glucose with (alternatively called mixotrophically) or without (alternatively called heterotrophically) light^8^,^19^. In our previous reports, *C. zofingiensis* has been shown to be promising for producing biodiesel and the secondary ketocarotenoid astaxanthin^20^,^21^,^22^,^23^. The aim of the present study was to investigate the effect of light on glucose-fed *C. zofingiensis* cells with respect to lipid yield, cell division, carbon flux to lipid and starch, and the transcriptional expression of genes encoding enzymes involved in lipid synthesis.

## Results

### Growth characteristics of glucose-fed *C. zofingiensis* with or without light

**Figure 1:**
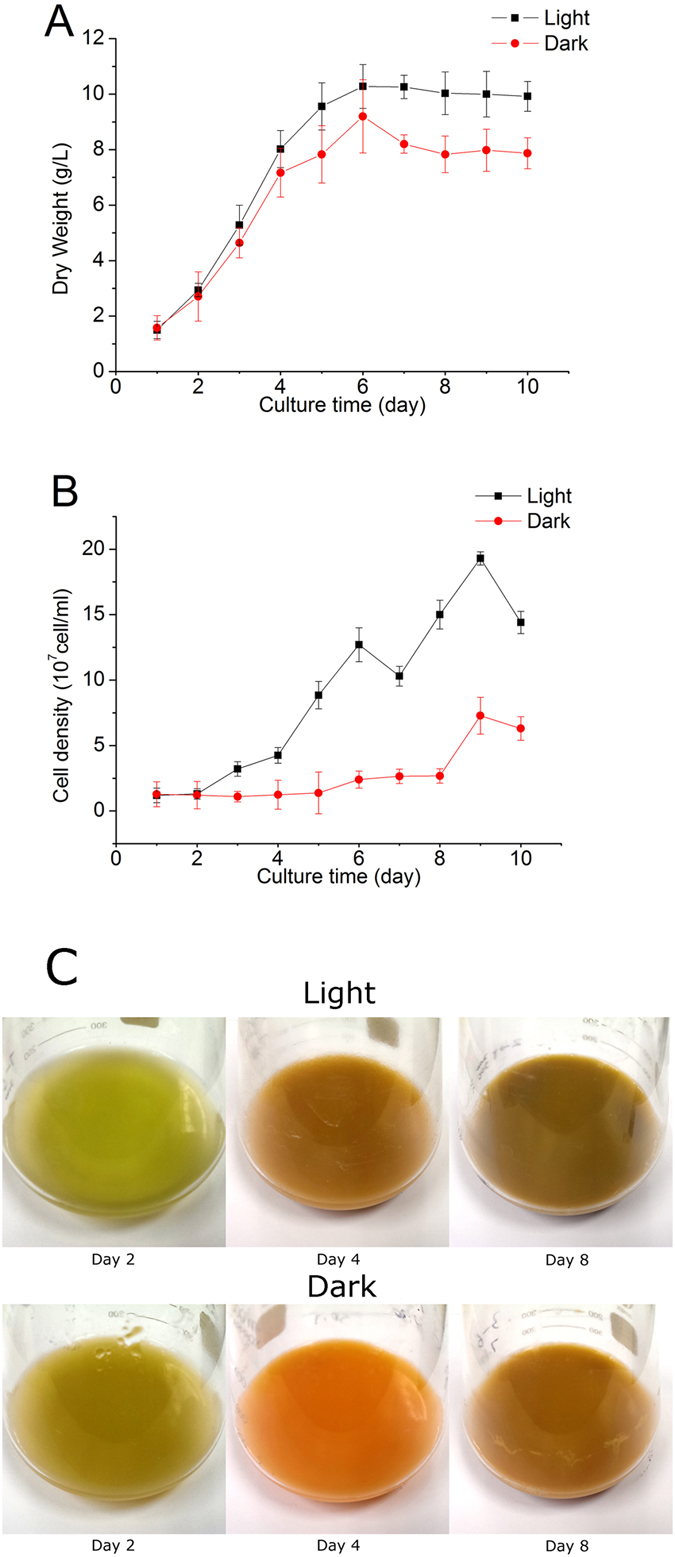
Time course of dry weight of *C. zofingiensis* cells with light and without light (A). Changes in culture color on the second, fourth, and eighth day with light and without light (**B**).

*C. zofingiensis* was cultured in the medium containing 30 g/L glucose, illuminated either with or without light. As indicated by Fig. 1, *C. zofingiensis* reached stationary growth phase after six days of cultivation under both culture conditions. Notably, in the presence of light, *C. zofingiensis* grew faster with a specific growth rate of 0.502 h^−1^, which is slightly higher than that without light (0.486 h^−1^) (Fig. 1A). Accordingly, *C. zofingiensis* with light gave a higher maximum dry cell weight (Fig. 1A). *C. zofingiensis* tended to turn yellow-orange-red during cultivation period (Fig. 1B), attributed to the synthesis and accumulation of secondary carotenoids, astaxanthin in particular^19^,^24^. It is worth noting that, *C. zofingiensis* also exhibited difference in the color of cultures between both conditions (Fig. 1B). The more intensive color of the light cultures resulted from the biosynthesis of light-induced chlorophylls.

### Light attenuates lipid accumulation while stimulating starch biosynthesis

**Figure 2:**
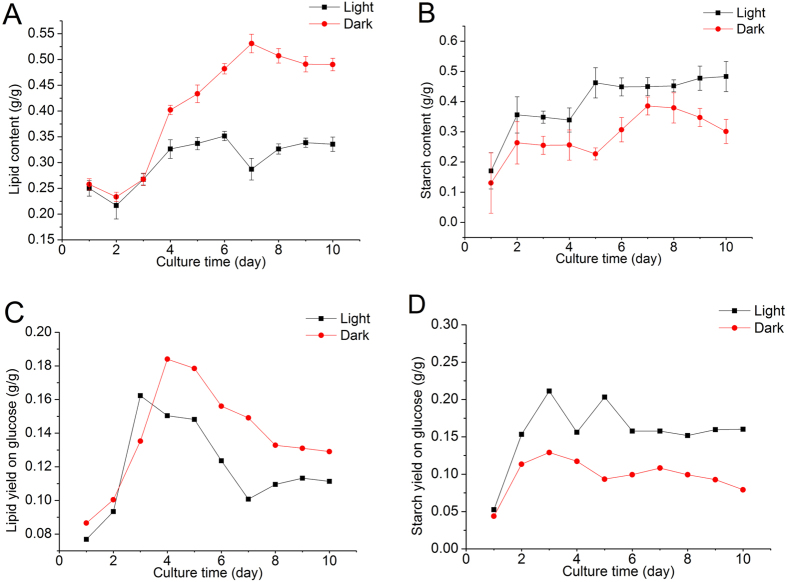
Time course of lipid content (A), starch content (B), lipid yield on glucose (C), and starch yield on glucose (D) in *C. zofingiensis* cells grown on glucose with or without light.

In green microalgae, lipid and starch biosyntheses share the common carbon precursors, though the regulation of carbon partitioning into these two biosynthetic pathways is not well understood^25^,^26^. Glucose-fed *C. zofingiensis* cultures maintained a basal lipid level during the first 3 days of cultivation; thereafter, the lipid built up rapidly and reached the maximal content of 53% of dry weight on day 7 (Fig. 2A). The algal cultures provided with light followed the similar pattern of lipid accumulation, but the lipid content was greatly attenuated as compared to the light-free cultures, e.g., 28% of dry weight on day 7, which is 48% less than the cultures without light. In contrast, there was greater starch accumulation in the cultures with light than in the cultures without light, and the starch content of the former remained higher than the latter during the whole cultivation period (Fig. 2B). Interestingly, starch content started to accumulate ahead of lipid accumulation (Fig. 2A,B). Yield on glucose can reflect the carbon flux allocation. Notably, opposite trend of flux to lipid and starch was observed in cells with and without light (Fig. 2C,D). As compared to the cultures with light, *C. zofingiensis* without light had higher lipid yield on glucose (Fig. 2C), though both of them dropped after cells started to divide. On the contrary, starch yield on glucose with light was higher than that without light during whole culture period (Fig. 2D). Different from lipid yield on glucose, starch yield remained relatively stable.

### Light-induced lipid reduction is accompanied by accelerated cell proliferation

**Figure 3:**
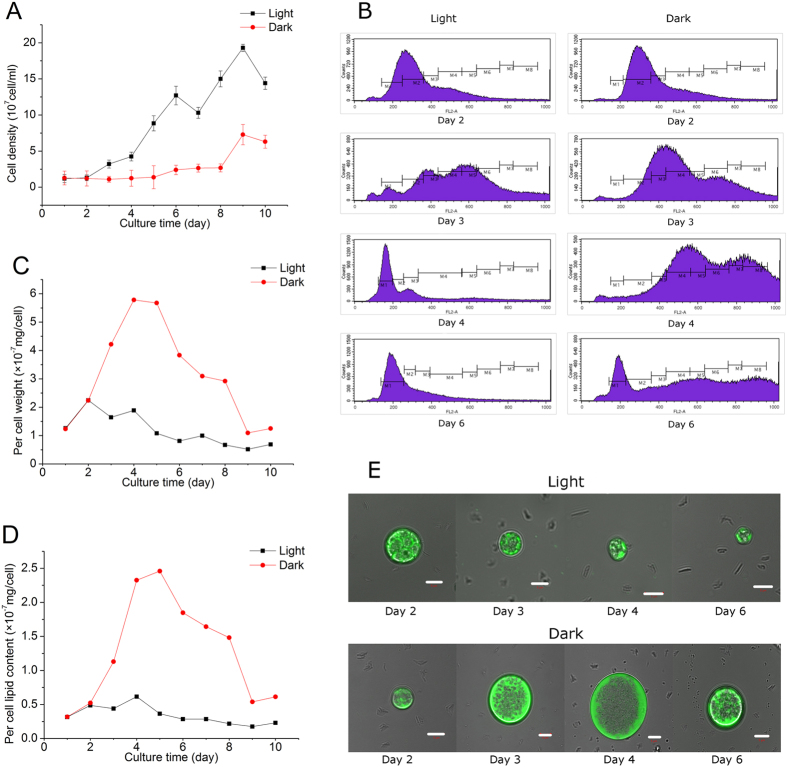
Time course of cell density (A), per cell weight (C), per cell lipid content (D) in *C. zofingiensis* grown with light and without light. Cell cycle determined by flow cytometry (**B**) with labels of M1, M2 and M3 representing G1/G0 phase, S phase and G2/M phases, respectively, and M4 to M8 representing polyploid phases. BODIPY stained intracellular lipids (**E**) of *C. zofingiensis* cells grown on glucose with or without light. Bars in E represent 5 μm.

Figure 3A shows the time course of algal cell density grown with or without light. Dark-grown *C. zofingiensis* exhibited almost no change in cell number until day 6; in contrast, in the presence of light the alga started to divide on day 2 and reached up to be 15-fold higher in cell density, indicating that cell proliferation was greatly enhanced by light. Consistently, our cell cycle analysis data by flow cytometry also demonstrated that light promoted the algal cell mitosis (Fig. 3B). G1/G0 phase stands for cell in diploid or stationary form, which represents newborn cell after division. Obviously, light cultures showed a much earlier peak in G1/G0 phase than dark cultures (labeled as M1 in Fig. 3B).

There was an increase in the per cell weight observed during the early culture period, followed by a significant drop close to the initial value (Fig. 3C). Overall, *C. zofingiensis* without light maintained greater (up to 5 times) per cell weight than that with light. Similar to the pattern in per cell weight, the per cell lipid content of *C. zofingiensis* without light showed a drastic increase and reached the maximal value of 250 pg/cell after 5 days of cultivation, which is 5-fold higher than the light-illuminated cultures (Fig. 3D). In addition, BODIPY 505/515, a fluorescent lipophilic dye for the neutral lipids’ staining^27^, was employed to monitor the *in vivo* dynamic changes of lipids in *C. zofingiensis* cells (Fig. 3E). The green signals represent the staining of neutral lipids, predominantly in the form of TAGs. In accordance with the per cell weight (Fig. 3C) and lipid content (Fig. 3D), dark-grown *C. zofingiensis* exhibited a drastic increase in both cell size and florescent staining with a peak value obtained on day 4, while the algal culture with light reached the maximum on day 2, followed by a gradual decline in cell size and staining due likely to the accelerated cell division (Fig. 3E).

### Light alters the transcriptional expression of fatty acid biosynthetic genes

**Figure 4:**
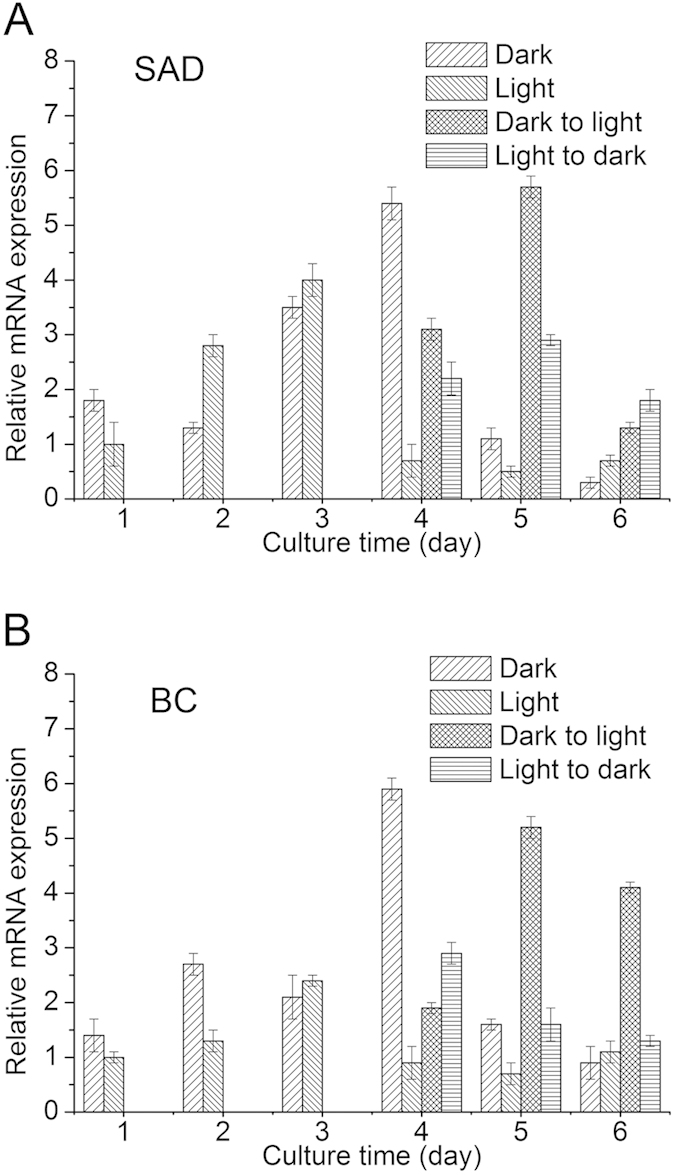
Relative transcript levels of *SAD* (A) and *BC* (B) in *C. zofingiensis* grown in four culture conditions: Dark culture, light culture, conversion from dark to light after three days and conversion from light to dark after three days; *SAD*, stearoyl ACP desaturase; *BC*, biotin carboxylase. The levels of gene expression were normalized relative to the cultures with light on day 1, which was set to 1.

It is commonly agreed that green algae follow the similar lipid biosynthetic pathway as in higher plants. Among the enzymes involved in lipid biosynthesis, acetyl-CoA carboxylase (ACCase) is a rate-limiting enzyme catalyzing the first committed step for *de novo* fatty acid synthesis in chloroplast^28^. Chloroplastic ACCase is composed of four subunits and the expression of the genes coding the subunits is autoregulated to each other. Thus, the characterization of one subunit such as biotin carboxylase (BC) can be representative of ACCase. Stearoyl ACP desaturase (SAD) introduces the first double bond to acyl chain and plays an important role in determining the degree of saturation of fatty acids^28^. To investigate the effect of light on fatty acid biosynthesis, the transcript levels of *SAD* and *BC* in *C. zofingiensis* were determined using a real time-PCR approach. In the dark-grown *C. zofingiensis* cells, an increase in the steady-state mRNA level of both *SAD* and *BC* was observed and the mRNA levels reached their maximum on day 4, much higher than the maximum values in light-grown cells on day 3 (Fig. 4A,B). This is well consistent with the data that dark cells showed a sharp increase in per cell lipid content on day 4 (Fig. 3C). The introduction of light to dark culture after 3 days of cultivation exerted a negative effect and attenuated the *SAD* and *BC* transcripts dramatically compared to dark-grown cells on day 4. It’s worth noting, however, that the expression of both genes increased sharply on day 5 and then decreased. When the light-illuminated cells transferred to dark, both *SAD* and *BC* transcriptional levels exhibited significantly higher than those in light-grown cells from day 4 to 6. Overall, *C. zofingiensis* accumulated more *SAD* and *BC* transcripts in dark than under light, which may explain why dark cells accumulated more lipids than light cells.

### Culture conditions have little effect on fatty acid profiles

**Figure 5:**
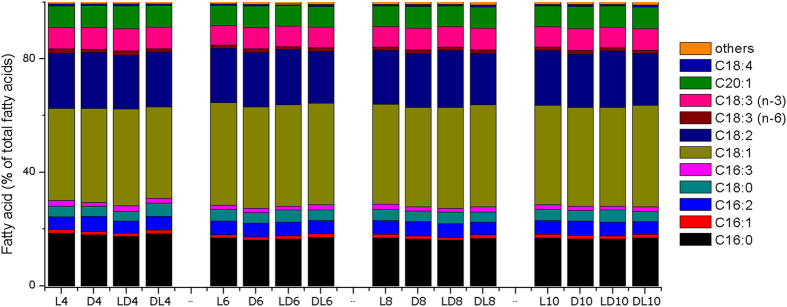
Fatty acid profiles of *C. zofingiensis* lipids in four culture conditions: L (light culture), D (dark culture), LD (conversion from light to dark after three days), and DL (conversion from dark to light after three days); The numbers following the letters represent culture days. Data are expressed as percentages of total fatty acids.

The quality of biodiesel is largely determined by its fatty acid composition^29^. GC-MS was employed to analyze the fatty acid profiles in *C. zofingiensis* under different culture conditions. The algal cells produced fatty acids mainly in the form of C18:1 (32.2%–35.8%), C18:2 (18.2%–20.1%), and C16:0 (16.1%–18.5%), which together account for more than 66% of total fatty acids, regardless of the culture conditions (Fig. 5). Although the total lipid contents varied greatly, no significant difference was observed in the fatty acid composition under the tested conditions (Fig. 5).

## Discussion

*C. zofingiensis* can grow well photoautotrophically, mixotrophically, and heterotrophically. Under heterotrophical growth conditions, organic carbon sources, glucose in particular, are the sole carbon and energy sources^8^,^19^,^24^. *Chlorella* possesses an inducible hexose/H^+^ active symport system that is responsible for the uptake of glucose from the medium^30^,^31^. In the presence of glucose, the hexose/H^+^ symport system protein in *Chlorella* cells can be activated in just a few minutes^32^. Usually, the specific growth rate of glucose-fed microalgae growing with light is approximately the sum of cell growth rates under photoautotrophic and glucose-fed conditions in dark^33^. This might explain our findings that *C. zofingiensis* grew faster feeding on glucose in the presence of light (Fig. 1A). In green microalgae, lipid and starch are the two dominant energy storage forms and share the common carbon precursors for biosynthesis. It has been reported that photoautotrophic *Chlorella* is able to accumulate lipid and starch up to 60% and 45% of dry weight, respectively, depending on the algal strains and culture conditions^34^,^35^,^36^,^37^,^38^. Little attention, however, has been paid to the effect of light on carbon flux to lipid and starch in algal cells feeding on organic carbon sources such as glucose. In the present study, for the first time, we investigated the accumulation of lipid and starch in glucose-fed *C. zofingiensis* with or without light. Both lipid and starch contents increased, but starch accumulation preceded lipid synthesis (Fig. 2A,B), which is consistent with the previous studies in photoautotrophically cultured *C. zofingiensis*^39^ and *Pseudochlorococcum* sp^40^. Compared to the light cultures, the dark cultures tended to accumulate more lipids and less starch (Fig. 2). One possible explanation is that lipids require more reducing power than starch for production, while glucose-fed cultures in dark can generate more reducing power^17^. For example, lipid synthesis for a C18 fatty acid needs 16 NADPH molecules^33^, which is less energetically economical than starch synthesis, as the latter requires 6 NADPH molecules and 9 ATP molecules to form an 18-carbon molecule^40^. On the other hand, light can stimulate algae proliferation (Fig. 3). Cell division is an energy-consuming process and microalgae tend to accumulate sufficient energy before proliferation. The energy storage materials, lipids and starch in particular, tend to accumulate in the algal cells under stress conditions when the cell growth halts. Lenneke^41^ discovered that starch and lipid accumulation in *Neochloris oleoabundans* occurred before mitosis. Stress relief facilitates the degradation of lipids or starch, which can provide energy for boosting the cell growth. In the present study, we noticed a sharp decrease in lipid yield on glucose but not in starch yield on glucose after cell proliferation (Fig. 2C,D), suggesting that glucose-fed C. *zofingiensis* cells tended to utilize lipids rather than starch to provide energy for cell division.

*SAD* and *BC* are two genes encoding key enzymes involved in fatty acid biosynthesis. It has been suggested that the control of these two genes on fatty acid biosynthesis may occur at transcriptional level in *C. zofingiensis*^25^. Consistent with the attenuated lipid yield on glucose after 3 days (Fig. 2C), a depressed expression level of these two genes was found in the cells provided with light, as compared to the dark-grown cells on day 4 (Fig. 4A,B). In this context, light might down-regulate the expression of fatty acid biosynthetic genes, leading to the decrease in lipid content to provide energy for cell proliferation, which would direct more carbon flux from glucose to starch biosynthetic pathway resulting in enhanced starch accumulation.

Fatty acid composition determines the quality of biodiesel and is subject to change in different culture conditions. The fatty acids in *C. zofingiensis* cells consisted predominantly of 16–18 carbons and the maximum unsaturation degree was 3, which are similar to that of plant oils currently used for biodiesel production^42^. It was found that oil from *C. zofingiensis* in dark was more suitable than that from photoautotrophic cells for biodiesel production, as the former contained high content of oleic acid (C18:1), linoleic acid (C18:2), and palmitic oil (C16:0), which can balance oxidative stability and low-temperature properties and can promote the quality of biodiesel^8^. Notably, our result suggested that light had no significant effect on fatty acid composition in glucose-fed *C. zofingiensis.*

*C. zofingiensis* accumulates lipid efficiently in the dark supplemented with glucose. Light has a dual effect on *C. zofingiensis*: promoting cell proliferation and biomass yield, while on the other hand enhancing starch accumulation at the cost of lipid possibly through the inhibition of lipid biosynthetic pathway. Our results provided insights into utilizing different culture conditions for boosting biomass, lipid content, and lipid yield on glucose.

## Methods

### Microalgal strain and culture conditions

*C. zofingiensis* (ATCC 30412) was obtained from the American Type Culture Collection (ATCC, Rockville, MD, USA). This alga was maintained at 4 °C on an agar slant containing Kuhl medium^20^, consisting of (per liter) 1.01 g KNO_3_; 0.62 g NaH_2_PO_4_·H_2_O; 0.089 g Na_2_HPO_4_·2H_2_O; 0.247 g MgSO_4_·7H_2_O; 14.7 mg CaCl_2_·2H_2_O; 6.95 mg FeSO_4_·7H_2_O; 0.061 mg H_3_BO_3_; 0.169 mg MnSO_4_·H_2_O; 0.287 mg ZnSO_4_·7H_2_O; 0.0025 mg CuSO_4_·5H_2_O; and 0.01235 mg (NH_4_)_6_Mo_7_O_24_·4H_2_O. Thirty grams of glucose was added to 1 liter of medium. The pH of the medium was adjusted to pH 6.1 prior to autoclaving. Briefly, 10 mL of liquid Kuhl medium was inoculated with cells from slants, and the alga was grown aerobically in flasks at 25 °C for 4 days with orbital shaking at 150 rpm and with continuous illumination at 50 μmol photon m^−2^·s^−1^. The cells were then inoculated at 10% (v/v) into a 250-mL Erlenmeyer flask containing 50 mL of the growth medium. Algal cells in exponential growth phase were used as seed cells for the following batch cultures.

For dark cultivation, seed cells were inoculated into 100 mL of fresh medium in 500-mL flasks at a starting cell density of 0.5 g/L and were grown in the dark at 25 °C with orbital shaking at 150 rpm. Light cultivation was conducted under continuous illumination at 50 μmol photon m^−2^·s^−1^; the other parameters were the same as for dark culture.

A total of 48 samples were divided into four groups, which were cultivated in the dark, in light, in dark-to-light and in light-to-dark conditions. Samples in the latter 2 groups were transferred to the light or dark after three days of incubation. Samples from four culture condition groups were collected for testing every day.

### Analysis of lipid content and starch content

Total lipids were extracted from lyophilized cell powder according to Converti^43^ with some modifications. A 100-mg mass was ground before 2 extractions with petroleum ether; the supernatants were then merged and evaporated with N_2_. The crude oil was then weighed. Lipid content was expressed as lipid weight per unit biomass.

The starch content was analyzed using the above defatted sediment according to a modified method used by Brányiková^34^. A 30% perchloric acid solution was added to 5 mg of sediment, stirred for 15 min at 25 °C and centrifuged. This procedure was repeated 3 times. The extracts were combined, and the volume was adjusted to 10 mL. Next, 2-mL aliquots of solubilized starch solution were reacted with 5 mL of concentrated sulfuric acid (98% by weight) and 1 mL of phenol (6%, w/v) at room temperature for 10 min. The absorbance was read in a spectrophotometer at 490 nm. Samples were then quantified by comparison to a calibration curve using glucose as the standard. Starch content was expressed as starch weight per unit biomass.

### Determination of cell density, biomass, specific growth rate, per cell weight, per cell lipid content, lipid and starch yield on glucose

Cell density were counted using a hemocytometer. Microalgal cells were centrifuged and filtered through a pre-dried Whatman GF/C filter (Cat No 1822-047) after 2 washes with distilled water. Next, the filter paper was dried at 80 °C in a vacuum oven for 12 h and was subsequently cooled down to room temperature before weighing. The biomass was expressed as cell dry weight. The specific growth rate (μ_max_) was calculated according to:


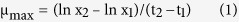


where x_2_ and x_1_ are the dry weight (g/L) at time t_2_ and t_1_, respectively.

Per cell weight was calculated as biomass divided by cell density. Per cell lipid content was calculated according to:


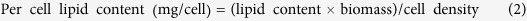


Glucose concentration in the supernatant was determined according to Miller^44^ and utilized glucose was calculated as initial glucose concentration minus glucose concentration in the supernatant.

Lipid and starch yield on glucose were calculated according to:


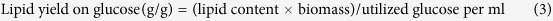



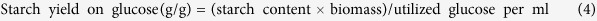


### Fatty acid analysis

Lyophilized algal powder (20 mg) was incubated overnight in a solvent mixture (1 mL toluene, 2 mL 1% sulphuric acid in methanol and 0.8 mg heptadecanoic acid in 0.8 mL hexane as the internal standard) at 50 °C for the formation of fatty acid methyl esters (FAMEs) by transesterification. FAMEs were then extracted three times with hexane in a reciprocating shaker (MS3, IKA, Germany). The FAMEs were analyzed by using a GC-MS-QP 2010 SE (Electron Ionization type) gas chromatograph-mass spectrometer (SHIMADZU, Japan) and a Stabilwax-DA capillary column (30 m × 0.25 mm × 0.25 μm) (SHIMADZU, Japan). Helium was used as the carrier gas. The injection temperature, ion temperature and interface temperature were set at 250 °C, 200 °C and 260 °C, respectively. The initial column temperature was set at 150 °C. The column temperature subsequently rose to 200 °C at 10 °C/min and then to 250 °C at 15 °C/min, followed by a hold at 250 °C for 3 min. FAMEs were identified using the NIST 11 mass spectral library (NIST/EPA/NIH mass spectral library, 2011 edition). The quantities of individual FAMEs were calculated by their peak areas according to the total ion chromatogram (TIC), using heptadecanoic acid as the internal standard.

### BODIPY staining and laser scanning confocal microscope (LSCM) imaging

The lipophilic fluorescent dye BODIPY 505/515 (Invitrogen Molecular Probes, Carlsbad, CA, USA) was used to monitor lipid storage in algal cells according to Cooper^45^ with minor modifications. Briefly, a 5-mM stock solution of BODIPY 505/515 made by dissolving the dye in anhydrous dimethyl sulfoxide (DMSO) was added directly to the algal suspensions to achieve a final concentration of 5 μM. The cells were then observed and recorded with an LSM 710 NLO & DuoScan System (Zeiss, Germany) after being dyed for 10 min in the dark.

### RNA isolation and real-time RT-PCR assay

The expression levels of two genes involved in fatty acid synthesis were determined using real-time PCR according to Liu^46^. Briefly, RNA was isolated from aliquots of approximately 10^8^ cells using TRIZOL reagent (Molecular Research Center, Cincinnati, OH, USA) according to the manufacturer’s instructions. The concentration of total RNA was determined spectrophotometrically at 260 nm. Total RNA (1 μg) extracted from different samples was reverse transcribed to cDNA using the SuperScript III First-Strand Synthesis System (Invitrogen, Carlsbad, CA, USA) for reverse transcription PCR (RT-PCR) primed with oligo(dT) according to the manufacturer’s instructions. Real-time RT-PCR analysis was performed using 1 μL of the RT reaction mixture in a total volume of 20 μL with specific primers and the Platinum SYBR Green qPCR SuperMix-UDG (Invitrogen). PCR amplification was conducted using specific primers targeting BC (forward, 5′-GTGCGATTGGGTATGTGGGGGTG-3′ and reverse, 5′-CGACCAGGACCAGGGCGGAAAT-3′), SAD (forward, 5′-TCCAGGAACGTGCCACCAAG-3′ and reverse, 5′-GCGCCCTGTCTTGCCCTCATG-3′), and the internal control actin (ACT) gene(forward, 5′-TGCCGAGCGTGAAATTGTGAG-3′ and reverse, 5′-CGTGAATGCCAGCAGCCTCCA-3′). PCR was performed in a Bio-Rad iCycler IQ Multi-Color Real-Time PCR Detection System (Bio-Rad, Hercules, CA). The relative levels of the amplified mRNAs were evaluated using the 2^−ΔΔCt^ method^47^, using the actin gene for normalization.

### Cell cycle analysis

Cell cycle was determined by flow cytometry (FCM) with prodium iodide (PI) staining. The method was according to Gerashchenko^48^ with modifications. Briefly, 10^6^ cells were collected and washed twice with PBS. Then methanol was added in before removing PBS. Cells in methanol were dispersed and stored in 4 °C for analysis. Upon analysis, the samples were washed with PBS and then stained with 50 lg/ml PI in the presence of 25 lg/ml RNase A in 37 °C bath for 30 min. The cell cycle distribution of 10,000 cells was recorded by a flow cytometer (BD FACS Calibur), and result was analyzed with ModFit software.

## Additional Information

**How to cite this article**: Chen, T. *et al.* Light attenuates lipid accumulation while enhancing cell proliferation and starch synthesis in the glucose-fed oleaginous microalga *Chlorella zofingiensis*. *Sci. Rep.* **5**, 14936; doi: 10.1038/srep14936 (2015).
